# High-efficiency direct methane conversion to oxygenates on a cerium dioxide nanowires supported rhodium single-atom catalyst

**DOI:** 10.1038/s41467-020-14742-x

**Published:** 2020-02-19

**Authors:** Shuxing Bai, Fangfang Liu, Bolong Huang, Fan Li, Haiping Lin, Tong Wu, Mingzi Sun, Jianbo Wu, Qi Shao, Yong Xu, Xiaoqing Huang

**Affiliations:** 1 College of Chemistry, Chemical Engineering and Materials Science, Soochow University, Jiangsu, 215123 China; 2 Institute of Functional Nano&Soft Materials (FUNSOM), Jiangsu Key Laboratory for Carbon-Based Functional Materials & Devices, Soochow University, Jiangsu, 215123 China; 3 Department of Applied Biology and Chemical Technology, The Hong Kong Polytechnic University, Hung Hom, Kowloon, Hong Kong SAR China; 4 State Key Laboratory of Metal Matrix Composites, School of Materials Science and Engineering, Shanghai Jiao Tong University, Shanghai, 200240 China; 5 Center of Hydrogen Science, Shanghai Jiao Tong University, Shanghai, 200240 China

**Keywords:** Heterogeneous catalysis, Density functional theory, Nanowires

## Abstract

Direct methane conversion (DMC) to high value-added products is of significant importance for the effective utilization of CH_4_ to combat the energy crisis. However, there are ongoing challenges in DMC associated with the selective C−H activation of CH_4_. The quest for high-efficiency catalysts for this process is limited by the current drawbacks including poor activity and low selectivity. Here we show a cerium dioxide (CeO_2_) nanowires supported rhodium (Rh) single-atom (SAs Rh-CeO_2_ NWs) that can serve as a high-efficiency catalyst for DMC to oxygenates (i.e., CH_3_OH and CH_3_OOH) under mild conditions. Compared to Rh/CeO_2_ nanowires (Rh clusters) prepared by a conventional wet-impregnation method, CeO_2_ nanowires supported Rh single-atom exhibits 6.5 times higher of the oxygenates yield (1231.7 vs. 189.4 mmol g_Rh_^−1^ h^−1^), which largely outperforms that of the reported catalysts in the same class. This work demonstrates a highly efficient DMC process and promotes the research on Rh single-atom catalysts in heterogeneous catalysis.

## Abstract (web summary)

Direct methane conversion to high value-added products is a promising way for highly-efficient utilization of methane. Here, the authors demonstrate that rhodium single-atom supported on cerium dioxide nanowires can selectively convert methane to oxygenates under mild conditions.

## Introduction

Methane (CH_4_) is in fact among the most important and attractive feedstocks for producing methanol (CH_3_OH) and other high value-added products in the chemical industry^1,2^. In the traditional processes, CH_4_ is converted into CH_3_OH indirectly via the formation of syngas (H_2_ and CO), which is an energy-hungry process that needs to be performed under high temperature^3,4^. Therefore, the direct CH_4_ conversion (DMC) to CH_3_OH, which is regarded as a “dream reaction” in chemical industry, has been the subject of intensive study for decades^5–7^. For instance, Periana et al. reported that CH_4_ can be converted to CH_3_OH by mercuric ions in the presence of concentrated sulfuric acid^8^. Sushkevich et al.^9^ synthesized a copper-containing zeolite catalyst and used it for the conversion of CH_4_ to CH_3_OH with high selectivity (~ 97%) at 200 °C. More recently, Agarwal et al. used colloidal gold–palladium nanoparticles (Au–Pd NPs) to catalyze the DMC to CH_3_OH, methylhydroperoxide (CH_3_OOH), and formic acid (HCOOH) in the presence of hydrogen peroxide (H_2_O_2_) and oxygen (O_2_)^10^. The yield of these primary oxygenates product reaches ~53.6 mol kg_cat_^−1^ h^−1^ at a selectivity of 88.0% at 50 °C^10^. Shan et al.^2^ reported that mononuclear rhodium species on ZSM-5 can catalyze DMC to CH_3_OH and acetic acid (CH_3_COOH) using O_2_ and carbon monoxide (CO) with the total yield of oxygenates ~10 mmol g_cat._^−1^ h^−1^ and the selectivity of CO_2_ ~ 15%^2^. Despite the tremendous progress on the development of catalysts and technologies, DMC to oxygenates is extremely challenging because the selective activation of C−H bonds in CH_4_ under mild conditions is a tough issue. It is thus highly desired to develop active and selective catalysts for the DMC to oxygenates.

Noble metals-based single-atom catalysts (SACs) have emerged as a new frontier in heterogenous catalysis because of large ratio of surface atoms, low-coordination environment of metal centers, and strong metal–support interactions^11–13^. They have been widely studied in diverse processes with superior catalytic performance, including CO oxidation, CH_4_ conversion, oxygen reduction, water gas shift reaction, and so on^14–16^. Recent investigations show that SACs can be used as highly active and selective catalysts for alkynes hydrogenation, in which the isolated active sites are geometrically in favor of the selective hydrogenation of alkynes^17–20^. For instance, Kyriakou et al.^17^ demonstrated that the isolated Pd atoms in a Cu surface can be used as a highly selective catalyst for the hydrogenation of styrene and acetylene as compared with pure Cu or Pd metal. Yan et al.^18^ claimed that Pd SAs can selectively catalyze butadiene to butene because of the mono-π-adsorption mode and the steric effect induced by butadiene adsorption on the isolated Pd atoms. Typical for CH_4_ conversion, Tang et al.^15^ reported that the single-site Rh_1_O_5_ anchored in microporous aluminosilicates (ZSM-5) can catalyze the DMC to CH_3_COOH and CH_3_OH in the presence of CH_4_, CO, and O_2_ at ≤ 150 °C. It is found that the single-site Rh_1_O_5_ plays the role of the active site for DMC, while the rhodium oxide NPs on ZSM-5 are even not active for this transformation^15^. Kwon et al.^16^ demonstrated that Rh SA on ZrO_2_ can be used for the DMC to CH_3_OOH and CH_3_OH in H_2_O_2_ solution at 70 °C. However, the selectivity of oxygenates from Rh SA on ZrO_2_ is ~ 70% due to the different decomposition rate of H_2_O_2_ and CH_3_OOH on ZrO_2_^16^. Inspired by these reports, we believe that the Rh SACs can be used as promising catalysts for DMC, and the selectivity of oxygenates may be strongly related to the support for anchoring Rh SA.

Herein, we synthesized Rh-based SACs on CeO_2_ nanowires (SAs Rh-CeO_2_ NWs) via a simple hydrothermal process. The results show that SAs Rh-CeO_2_ NWs can be used as a highly efficient catalyst for DMC to oxygenates in the presence of H_2_O_2_ at 50 °C. Different from the previous work, our work reveals that the support for anchoring Rh SAs (i.e., CeO_2_ NWs) is involved in the formation of radicals, which can further enhance the activity of DMC. The total yield and selectivity of oxygenates reach ~1231.7 mmol g_Rh_^−1^ h^−1^ and 93.9%, respectively. To the best of our knowledge, the current DMC performance outperforms the reported catalysts in literatures. In situ characterizations and theoretical calculations show that CeO_2_ NWs play a vital role in the formation of ∙OOH and ∙OH radicals. SAs Rh-CeO_2_ NWs can selectively activate CH_4_ to ∙CH_3_, which further combines with ∙OOH and ∙OH radicals to form CH_3_OH and CH_3_OOH, respectively. By contrast, the Rh/CeO_2_ NWs tend to overoxidize CH_4_ to CO_x_ species with the assistance of ∙OH, leading to a low oxygenates’ yield and selectivity (189.4 mmol g_Rh_^−1^ h^−1^ and 56.4%).

## Results

### Preparation and morphology characterization

**Fig. 1:**
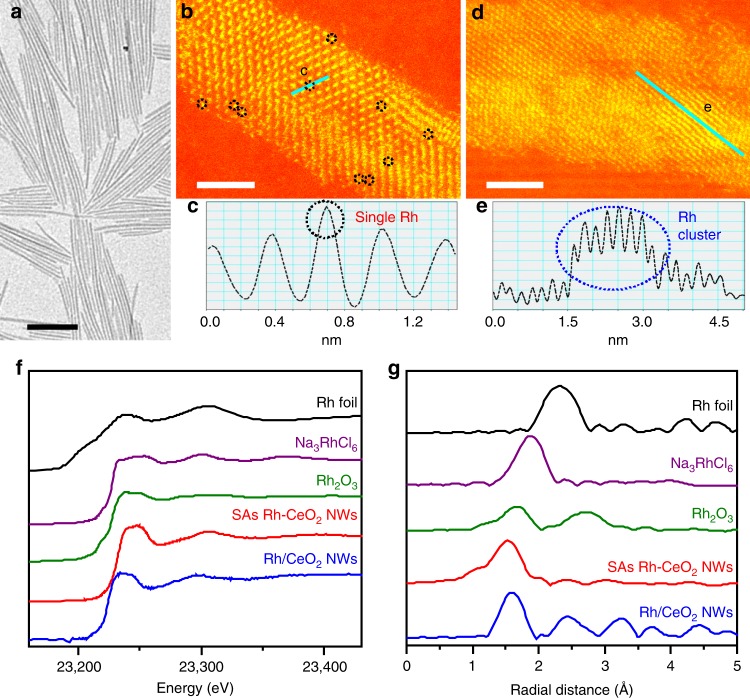
Structural analyses of SAs Rh-CeO_2_ NWs and Rh/CeO_2_ NWs. **a** TEM image of the SAs Rh-CeO_2_ NWs. **b** AC-HAADF/STEM image in temperature color of the SAs Rh-CeO_2_ NWs. The isolated Rh atoms are marked with black circles. **c** The intensity profile recorded from the line in panel (**b**). **d** AC-HAADF/STEM image in temperature color of the Rh/CeO_2_ NWs. The Rh cluster is marked with blue line. **e** The intensity profile recorded from the line in panel (**d**). **f**, **g** Rh K-edge XANES spectra (**f**) and Rh K-edge EXAFS spectra in *R* space (**g**) of the Rh foil, Na_3_RhCl_6_, Rh_2_O_3_, SAs Rh-CeO_2_ NWs, and Rh/CeO_2_ NWs. The scale bars in (**a**), (**b**), and (**d**) are 100, 2, and 2 nm, respectively.

CeO_2_ NWs were prepared via a simple hydrothermal process by adding cerium chloride (CeCl_3_), sodium oleate, deionized water (H_2_O), and *n*-butylamine into a stainless reactor. The synthetic method of SAs Rh-CeO_2_ NWs was the same as that of CeO_2_ NWs, except for adding additional sodium hexachlororhodate (Na_3_RhCl_6_). Rh/CeO_2_ NWs and Rh/CeO_2_-com were prepared by impregnating Na_3_RhCl_6_ on the as-prepared CeO_2_ NWs and commercial CeO_2_ via a conventional wet-impregnation method. The physicochemical properties are listed in Supplementary Table 1. Transmission electron microscopy (TEM) image shows that uniform CeO_2_ NWs with a diameter and length of ~ 6.2 nm and ~ 260 nm are obtained (Supplementary Fig. 1). No Rh nanoparticles are observed in TEM image, indicating that Rh atoms are well dispersed in SAs Rh-CeO_2_ NWs (Fig. 1a). The aberration-corrected high-angle annular dark-field scanning transmission electron microscopy (AC-HAADF/STEM) image in temperature color of SAs Rh-CeO_2_ NWs indicates Rh atoms are presented as SA state (Fig. 1b, c; Supplementary Fig. 2). By contrast, Rh clusters with the size of ~1.5 nm appear in the AC-HAADF/STEM image in temperature color of Rh/CeO_2_ NWs (Fig. 1d, e; Supplementary Fig. 3). In X-ray diffraction (XRD) pattern of SAs Rh-CeO_2_ NWs, only the characteristic peaks of CeO_2_ at 2*θ* = 28.5, 33.1, 47.5, 56.3, 69.4, 76.7, 79.1, and 88.4° (PDF No. 43-1002) are observed (Supplementary Fig. 4), indicating that Rh atoms are highly dispersed on CeO_2_ NWs and commercial CeO_2_, despite the full wavelength at half maximum (FWHM) of the characteristic peaks corresponding to CeO_2_ NWs is much broader than that of commercial CeO_2_ due to its small diameter of ~6.2 nm^21^. It should be noted that the absence of peaks corresponding to Rh clusters in the XRD pattern of Rh/CeO_2_ NWs might be attributed to the very small-sized Rh clusters and low content of Rh in the catalyst. X-ray photoelectron spectroscopy measurements were performed to characterize the chemical states of elements on the surface of CeO_2_ NWs, SAs Rh-CeO_2_ NWs, Rh/CeO_2_ NWs, and Rh/CeO_2_-com. As shown in Supplementary Fig. 5, it is found that Rh are in their oxidation state (Rh^3+^), while Ce consist of Ce^4+^ (881.8, 888.3, 897.6, 900.3, 907.2, and 916.0 eV) and Ce^3+^ (884.6 and 903.1 eV)^22^. The binding energy shifts of Rh in XPS spectra indicate that the different synergies among those catalysts. Compared with the Rh/CeO_2_-com (308.9 eV) and Rh/CeO_2_ NWs (309.1 eV), Rh 3d5/2 peak obviously shifts to 309.4 eV in SAs Rh-CeO_2_ NWs, suggesting the existence of electron transfer from Rh to CeO_2_ in SAs Rh-CeO_2_ NWs (Supplementary Fig. 5a). On the other hand, detailed peak fittings demonstrate that the proportions of Ce^3+^ are different among these samples (Supplementary Fig. 5b). In particular, the proportion of Ce^3+^ in Rh/CeO_2_-com is 12.4%, which is much lower than that in CeO_2_ NWs (16.0%), Rh/CeO_2_ NWs (16.5%), and SAs Rh-CeO_2_ NWs (16.7%). XPS results imply a much stronger synergy exists in SAs Rh-CeO_2_ NWs in comparison with Rh/CeO_2_ NWs and Rh/CeO_2_-com. No obvious peaks in Na 1 s and Cl 2p XPS spectra suggest that neglect amount of Na and Cl remain on catalysts (Supplementary Fig. 5c, d).

To study the electronic structures and coordination states of Rh in the SAs Rh-CeO_2_ NWs and the Rh/CeO_2_ NWs, X-ray absorption near-edge spectroscopy (XANES) and extended X-ray fine structure (EXAFS) were measured at Rh K-edge. Rh foil, Rh_2_O_3_, and Na_3_RhCl_6_ were used as references. Comparing with the edge position in the XANES spectra of references, Rh in SAs Rh-CeO_2_ NWs and Rh/CeO_2_ NWs are presented as their oxidation states (Fig. 1f). As shown in Fig. 1g, the Rh−Rh coordination, Rh−Cl coordination, Rh−O, and the second shell of Rh−O coordination are observed at ~2.3 Å, ~1.9 Å, ~1.7 Å, and ~2.7 Å, respectively^15^. For Rh/CeO_2_ NWs, Rh−O, and Rh−Rh coordination appear at ~1.6 Å and ~2.4 Å in the *R*-space EXAFS spectrum, indicating the presence of Rh clusters in Rh/CeO_2_ NWs. By contrast, only the feature of Rh−O coordination at ~1.6 Å is observed in the *R*-space spectrum of SAs Rh-CeO_2_ NWs (Fig. 1g). The disappearance of Rh−Rh coordination implies that Rh atoms in SAs Rh-CeO_2_ NWs are presented as isolated atoms, which is in good agreement with the observations in AC-HAADF/STEM image^16,23^. It is noted that no obvious Rh−Cl coordination is observed in the EXAFS spectrum of SAs Rh-CeO_2_ NWs, indicating that the Rh atoms are anchored by O atoms in CeO_2_ NWs via Rh−O coordination. In addition, the structures of SAs Rh-CeO_2_ NWs and Rh/CeO_2_ NWs were verified by the diffuse reflectance-infrared Fourier transform spectroscopy (DRIFTS) measurement using CO as a probe. As shown in Supplementary Fig. 6a, different from the previous reports that Rh SA gives geminal peaks in 2000−2100 cm^−1^, only one broad and weak peak is observed at 2000−2150 cm^−1^ in the CO-DRIFTS spectrum of SAs Rh-CeO_2_ NWs, which may be attributed to the low CO coverage on SAs Rh-CeO_2_ NWs^24^. The presence of oxidation species at 1250−1700 cm^−1^ indicates that the adsorbed CO molecules are oxidized, further confirming the low CO coverage on the surface of SAs Rh-CeO_2_ NWs. When SAs Rh-CeO_2_ NWs was pretreated in CO at 50 °C for 0.5 h, the intensity of peak at 2000−2150 cm^−1^ obviously increases, despite the appearance of oxidation species. Further increasing the pre-treatment temperature to 150 °C, two intense peaks appear at 2101 and 2030 cm^−1^ in CO-DRIFTS spectrum of SAs Rh-CeO_2_ NWs, which correspond to the symmetric and asymmetric vibration of gem-dicarbonyl doublet CO (i.e., Rh(CO)_2_) (Supplementary Fig. 6a, red curve)^25^. For Rh/CeO_2_ NWs, in addition to the peaks of oxidation species, multiple peaks corresponding to the symmetric (2101 cm^−1^) and asymmetric vibration (2030 cm^−1^) of Rh(CO)_2_ and CO linear adsorption on Rh^δ+^ (2133 cm^−1^) are observed in CO-DRIFTS spectrum (Supplementary Fig. 6b). When Rh/CeO_2_ NWs was pretreated at 50 °C and then 150 °C for 0.5 h, two peaks appear at 1860 and 2060 cm^−1^ in CO-DRIFTS spectrum, which correspond to the CO bridge adsorption and linear adsorption on Rh, respectively (Supplementary Fig. 6b, red curve)^24,25^. The absence of CO bridge adsorption in the CO-DRIFTS spectrum further confirms the structures of Rh SA in SAs Rh-CeO_2_ NWs, which is in good agreement with the result of AC-HAADF/STEM and X-ray absorption spectroscopy^25^.

### DMC performance of SAs Rh-CeO_2_ NWs and Rh/CeO_2_ NWs

**Fig. 2:**
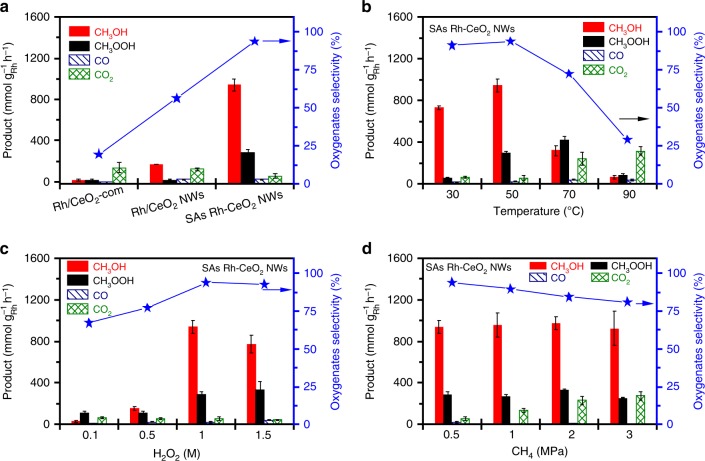
DMC performance on different catalysts. **a** The yield and selectivity of oxygenates from Rh/CeO_2_-com, Rh/CeO_2_ NWs, and SAs Rh-CeO_2_ NWs. Reaction conditions: *P*_CH4_: 0.5 MPa, H_2_O_2_: 20 mL (1 M), *T*: 50 °C, reaction time: 1 h, and catalyst weight: 10 mg. **b** DMC performance at different temperatures over SAs Rh-CeO_2_ NWs. Reaction conditions: *P*_CH4_: 0.5 MPa, H_2_O_2_: 20 mL (1 M), *T*: 30-90 °C, reaction time: 1 h, and catalyst weight: 10 mg. **c** DMC performance at different H_2_O_2_ concentrations over SAs Rh-CeO_2_ NWs. Reaction conditions: *P*_CH4_: 0.5 MPa, H_2_O_2_: 20 mL (0.1–1.5 M), *T*: 50 °C, reaction time: 1 h, and catalyst weight: 10 mg. **d** DMC performance at different CH_4_ partial pressure over SAs Rh-CeO_2_ NWs. Reaction conditions: *P*_CH4_: 0.5–3 MPa, H_2_O_2_: 20 mL (1 M), *T*: 50 °C, reaction time: 1 h, and catalyst weight: 10 mg. The error bars are defined as standard deviation of three experiments.

All the catalysts of Rh/CeO_2_-com, Rh/CeO_2_ NWs, and SAs Rh-CeO_2_ NWs were used for DMC in a pressurized reactor. Products were analyzed by gas chromatography and ^1^H nuclear magnetic resonance spectroscopy (^1^H-NMR). As shown in Fig. 2a, Rh/CeO_2_-com gives the CH_3_OH, CH_3_OOH, and CO_x_ yield of 17.6, 15.6, and 137.4 mmol g_Rh_^−1^ h^−1^, respectively. When Rh/CeO_2_ NWs are used as a catalyst, the yield of CH_3_OH and CH_3_OOH increase to 170.4 mmol g_Rh_^−1^ h^−1^ and 19.0 mmol g_Rh_^−1^ h^−1^, respectively, while the total yield of CO_x_ is 146.3 mmol g_Rh_^−1^ h^−1^. The low selectivities of oxygenate on Rh/CeO_2_-com (19.5%) and Rh/CeO_2_ NWs (56.4%) indicate that CH_4_ tends to be overoxidized into CO_x_ on Rh/CeO_2_-com and Rh/CeO_2_ NWs. By contrast, when SAs Rh-CeO_2_ NWs are used as a catalyst, the selectivity of CO_x_ significantly decreases to 6.1%, indicating that the overoxidation of CH_4_ is strongly suppressed on SAs Rh-CeO_2_ NWs. Correspondingly, the yield of CH_3_OH and CH_3_OOH significantly increase to 940.3 and 291.4 mmol g_Rh_^−1^ h^−1^, respectively. Moreover, SAs Rh-CeO_2_ NWs were tested for DMC under different conditions (i.e., temperature, H_2_O_2_ concentration, CH_4_ pressure, and catalysts amount). As depicted in Fig. 2b, it is found that the increase of temperature leads to a volcano-shape selectivity and yield of oxygenates. Typically, the selectivity and yield of oxygenates are 91.4% and 786.3 mmol g_Rh_^−1^ h^−1^ at 30 °C, which further increases to 93.9% and 1231.7 mmol g_Rh_^−1^ h^−1^ at 50 °C. Further increase in temperature will lead to a decrease in both the selectivity and yield of oxygenates. A similar tendency is observed when the concentration of H_2_O_2_ is increased from 0.1 to 1.5 M (Fig. 2c). For instance, the selectivity and yield of oxygenates are 67.1% and 145.0 mmol g_Rh_^−1^ h^−1^ at the H_2_O_2_ concentration of 0.1 M, which significantly increase to 93.9% and 1231.7 mmol g_Rh_^−1^ h^−1^ at the H_2_O_2_ concentration of 1.0 M, indicating H_2_O_2_ can significantly promote the DMC activity. It is noted that a further increase of H_2_O_2_ concentration to 1.5 M will result in a slight decay in oxygenates selectivity and yield. Furthermore, the effects of CH_4_ partial pressure on DMC performance were studied. As shown in Fig. 2d, the selectivity of CO_x_ strongly increases from 6.1% to 21.1% when the CH_4_ partial pressure is increased from 0.5 MPa to 3 MPa, despite the similar yield of oxygenates. We thus further tested the DMC performance by altering the weight of the catalyst, as shown in Supplementary Fig. 7. It is found that the yield of CH_3_OH and CH_3_OOH steadily increase as the increasing weight of catalyst, while the selectivity of CH_3_OH and CH_3_OOH are kept at ~94%. The positive effects of H_2_O_2_ concentration and catalyst weight but negligible effects of CH_4_ partial pressure on the oxygenates selectivity imply that Rh SA can efficiently activate C−H bonds in CH_4_ under the optimized conditions, and the rate-determining steps solely involve the reaction of CH_4_ activation intermediates with H_2_O_2_ (Fig. 2; Supplementary Fig. 7)^10^. In addition, SAs Rh-CeO_2_ NWs were used in ten consecutive DMC cycles to test the stability. As shown in Supplementary Fig. 8, no obvious decays in oxygenates selectivity and yield are observed after ten consecutive DMC cycles. The structures of Rh SA are reserved in the spent SAs Rh-CeO_2_ NWs, indicating that SAs Rh-CeO_2_ NWs can be used as a stable catalyst for DMC (Supplementary Fig. 9). Moreover, to the best of our knowledge, the current DMC performance has outperformed the reported catalysts (Supplementary Table 2), suggesting that SAs Rh-CeO_2_ NWs can be used as a highly efficient catalyst for DMC to oxygenates under mild conditions.

### Mechanistic studies on DMC

In order to study the mechanism of CH_4_ selective oxidation on SAs Rh-CeO_2_ NWs, DMC was performed using O_2_ as the oxidant to replace H_2_O_2_. As depicted in Supplementary Table 3, the selectivity of oxygenates is 5.9% when O_2_ is solely used as the oxidant, which significantly increases to 92.8% after the addition of 10 μmol H_2_O_2_. Correspondingly, the yield of oxygenates increases from 17.6 to 1159.4 mmol g_Rh_^−1^ h^−1^, indicating that H_2_O_2_ can significantly promote DMC to oxygenates. Based on the previous reports^10^, we speculated that the DMC in the presence of H_2_O_2_ might follow radical-triggered reaction paths. Therefore, electron paramagnetic resonance (EPR) was performed to detect the radicals in the present reaction system by using 5,5′-dimethyl-1-pyrroline-N-oxide (DMPO) as the radical scavenger. In order to label the radicals, two contrast experiments were performed in the systems of DMPO + H_2_O_2_ + Fe^2+^ and DMPO + H_2_O_2_ + Fe^2+^ + CH_3_OH (detailed information has been given in the experimental section). As shown in Supplementary Fig. 10, a four-line EPR spectrum with a relative peak ratio of 1:2:2:1 is obtained from the DMPO + H_2_O_2_ + Fe^2+^ system (Supplementary Fig. 10, green curve), which can be assigned to ∙OH radical^26^. For the system of DMPO + H_2_O_2_ + Fe^2+^ + CH_3_OH, a six-line EPR spectrum is recorded, which can be indexed as the characteristic peaks of ∙CH_3_ radical (Supplementary Fig. 10, blue curve)^26^. When DMPO is added into the reaction system, some new peaks of ∙OOH appear in the EPR spectrum in addition to those peaks of ∙CH_3_ and ∙OH radicals (Supplementary Fig. 10, red curve)^27^. The presence of ∙CH_3_, ∙OOH, and ∙OH radicals in the reaction system further confirms that the DMC on the SAs Rh-CeO_2_ NWs in H_2_O_2_ solution is triggered by radicals.

**Fig. 3:**
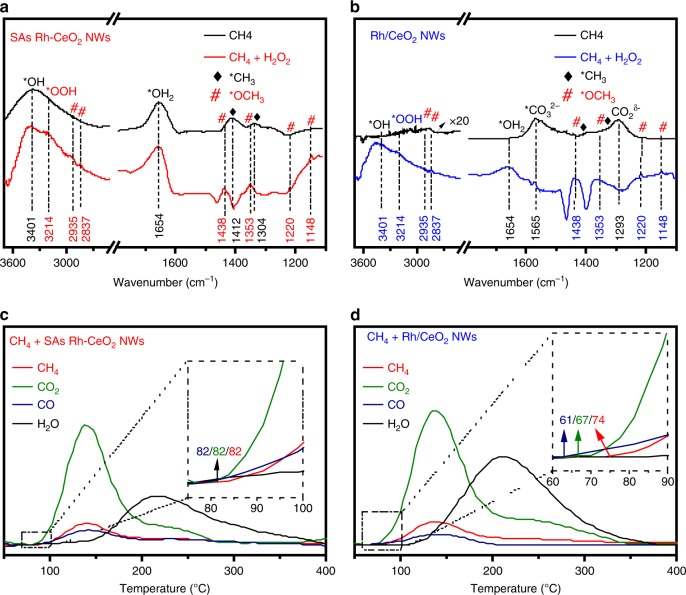
In situ CH_4_-DRIFTS and CH_4_-TPSR measurement. **a**, **b** In situ CH_4_-DRIFTS measurements on SAs Rh-CeO_2_ NWs (**a**) and Rh/CeO_2_ NWs (**b**). **c**, **d** CH_4_-TPSR measurements on SAs Rh-CeO_2_ NWs (**c**) and Rh/CeO_2_ NWs (**d**).

To further study the working mechanism of the radical-triggered DMC on SAs Rh-CeO_2_ NWs, in situ DRIFTS measurements were performed to analyze the surface species on SAs Rh-CeO_2_ NWs and Rh/CeO_2_ NWs when they were exposed to H_2_O_2_ and CH_4_. First, we exposed SAs Rh-CeO_2_ NWs and Rh/CeO_2_ NWs into H_2_O_2_. As shown in Supplementary Fig. 11, three intense peaks are observed at 3401, 3214, and 1654 cm^−1^ in the spectra of SAs Rh-CeO_2_ NWs and Rh/CeO_2_ NWs, which can be assigned to ^*^OH, ^*^OOH, and ^*^OH_2_, respectively^28^. Based on the observations in EPR spectra (Supplementary Fig. 10) and previous reports, we conclude that H_2_O_2_ can decompose into active radicals on CeO_2_ NWs^29,30^. When the SAs Rh-CeO_2_ NWs are exposed to CH_4_, the characteristic bands of ^*^CH_3_ appear at 1412 and 1304 cm^−1^ (see ref. ^31^), indicating that SAs Rh-CeO_2_ NWs can selectively activate CH_4_ into ^*^CH_3_ (Fig. 3a, black curve). Moreover, two additional bands are observed at 1635 and 3401 cm^−1^, which can be ascribed as ^*^OH_2_ and ^*^OH, respectively, suggesting that ^*^OH may combine with H from CH_4_ decomposition to form ^*^OH_2_. When SAs Rh-CeO_2_ NWs are exposed to the mixture of CH_4_ and H_2_O_2_ (Fig. 3a, red curve), new peaks appear at 2935, 2837, 1438, 1353, 1220, and 1148 cm^−1^ in the spectrum, which can be assign to the C−H asymmetric stretching vibration, C−H symmetric stretching vibration, CH_3_ scissoring vibration, CH_3_ asymmetric rocking vibration, C−O stretching vibration and CH_3_ symmetric rocking vibration of ^*^OCH_3_, respectively^32,33^. The appearance of ^*^OCH_3_, ^*^OH, and ^*^OOH demonstrates the formation of CH_3_OH and CH_3_OOH. By contrast, when Rh/CeO_2_ NWs are exposed to CH_4_, only two strong bands of CO_3_^2−^ and ^*^CO_2_^δ-^ are observed at 1589 and 1293 cm^−1^ (Fig. 3b, black spectrum)^34^. The absence of ^*^CH_3_, ^*^OH, and ^*^OH_2_ suggests that CH_4_ is overoxidized into CO_x_ species. When Rh/CeO_2_ NWs are exposed to the mixture of CH_4_ and H_2_O_2_, the intense band of CO_3_^2−^ in the spectrum implies that CO_x_ are the dominate products despite the appearance of ^*^OCH_3_ and ^*^OH bands (Fig. 3b, blue curve)^35^, which is consistent with our experimental results (Fig. 2a).

To further confirm the activation of CH_4_ on Rh, CH_4_ temperature programmed surface reaction (TPSR) was performed to investigate the CH_4_ conversion on CeO_2_ NWs, SAs Rh-CeO_2_ NWs, and Rh/CeO_2_ NWs. No obvious peaks are observed in the TPSR pattern of CeO_2_ NWs, suggesting that CeO_2_ NWs are inactive for CH_4_ activation under the indicated conditions (Supplementary Fig. 12). When CH_4_-TPSR measurements were performed on SAs Rh-CeO_2_ NWs and Rh/CeO_2_ NWs, the peaks of CH_4_, CO, CO_2_, and H_2_O appear in TPSR patterns, suggesting that Rh atoms play as the active sites for CH_4_ activation (Fig. 3c, d), which is in good agreement with results from the in situ DRIFTS measurement (Fig. 3a, b). Despite the appearance of peaks in TPSR patterns, the different onset temperatures (*T*_onset_) suggest that the reaction paths are different on SAs Rh-CeO_2_ NWs and Rh/CeO_2_ NWs (Fig. 3c, d). For SAs Rh-CeO_2_ NWs, the *T*_onset_ of CH_4_ (~82 °C, formed via ^*^CH_3_ hydrogenation) is closed to that of CO (~82 °C) and CO_2_ (~82 °C), indicating that ^*^CH_3_ hydrogenation (to form CH_4_) and overdehydrogenation (to form CO_x_) competitively occur on the SAs Rh-CeO_2_ NWs. By contrast, the *T*_onset_ of CO_2_ (~67 °C) and CO (~61 °C) is significantly lower than that of CH_4_ (~74 °C) in TPSR patterns of Rh/CeO_2_ NWs, indicating that overoxidation for the formation of CO and CO_2_ is preferential^5^.

### DFT calculations

**Fig. 4:**
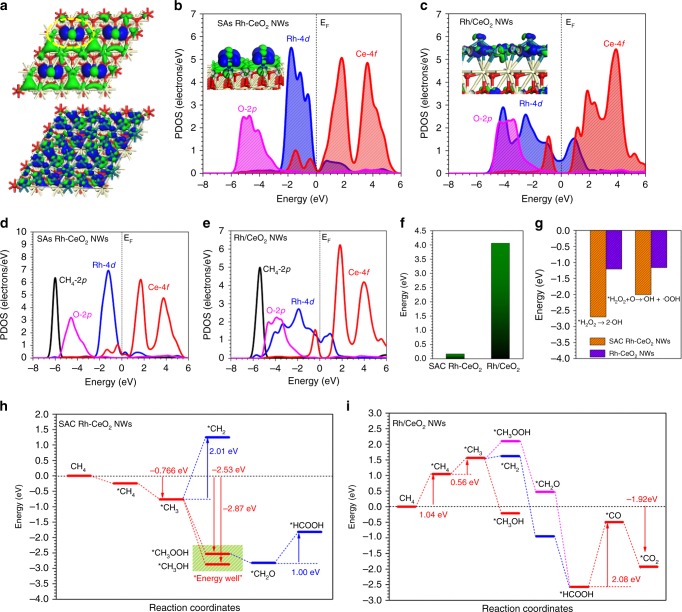
DFT calculations of DMC reaction paths on SAs Rh-CeO_2_ NWs and Rh/CeO_2_ NWs. **a** The real spatial contour plots for bonding and antibonding orbitals near E_F_ for SAs Rh-CeO_2_ NWs and Rh/CeO_2_ NWs. **b** The PDOS of SAs Rh-CeO_2_ NWs surface. **c** The PDOS of Rh/CeO_2_ NWs surface. **d** The PDOS of CH_4_ adsorption on SAs Rh-CeO_2_ NWs surface. **e** The PDOS of CH_4_ adsorption on Rh/CeO_2_ NWs surface. **f** Energy comparison of [O] desorption from CeO_2_ on SAs Rh-CeO_2_ NWs and Rh/CeO_2_ NWs. **g** The reaction energy comparison of generating radicals. **h** Reaction paths and energy profile of DMC over SAs Rh-CeO_2_ NWs. **i** Reaction paths and energy profile of DMC over the Rh/CeO_2_ NWs.

Finally, DFT calculations were performed to reveal the reaction paths of CH_4_ oxidation on SAs Rh-CeO_2_ NWs and Rh/CeO_2_ NWs. The real spatial orbital distribution apparently shows the concentrated electron-rich feature on SAs Rh, which indicates the Rh site possesses a superior reaction priority among the available sites on the surface. Meanwhile, Rh cluster on CeO_2_ shows widely separated electronic distribution over the surface, which shows the disperse electroactive region (Fig. 4a). The projected density of states (PDOS) support that the highly concentrated electronic activity of SAs Rh on the surfaces is attributed to the sharp Rh-4d occupation near the Fermi level (E_F_). The Ce-4f bands mainly locate above E_F_, while O-2p bands concentrate on E_v_ − 4.5 eV. The evident coupling between Rh-4d and Ce-4d confirms the stabilization of SAs Rh by the protection of bottom Ce (Fig. 4b). In comparison, the Rh-4d bands become much broad in Rh/CeO_2_, covering from E_v_ − 5.0 eV E_v_ + 2.0 eV. The evident match among Rh-4d bands and O-2p bands and Ce-4f bands indicates a strong coupling between the surface Rh cluster and bottom CeO_2_ (Fig. 4c). The electronic structures of CH_4_ adsorption are further presented to illustrate the different activity toward the DMC process. It is noted the dominant peak of Rh-4d in SAs Rh-CeO_2_ upshifts from E_v_ − 2.0 eV toward E_v_ − 1.0 eV due to the electron transfer with CH_4_ (Fig. 4d). Meanwhile, the close distance between Rh-4d bands and O-2p bands on Rh/CeO_2_ NWs demonstrates the strong couplings between CH_4_ and local O atoms, which lead to the over-binding effect with increased energy barriers for the consecutive dehydrogenation of CH_4_ (Fig. 4e).

Since EPR and in situ DRIFTS results show that lattice O atoms of CeO_2_ are feasibly bonded with H in H_2_O_2_. From the energetic view, DFT also proves the superior flexibility of O in SAs Rh-CeO_2_ NWs with only 0.16 eV energy barrier. Due to the steric hindrance from the surface coverage of Rh cluster, the energy barrier of the detachment of O in Rh/CeO_2_ NWs increases to 4.06 eV, demonstrating a slow efficiency of generating ∙OOH radicals (Fig. 4f). The further reaction energies also support that the generations of both ∙OH and ∙OOH radicals are much more preferred in SAs Rh-CeO_2_ NWs, satisfying the prerequisite of radical-mediated DMC process (Fig. 4g). The SAs Rh-CeO_2_ NWs deliver an overall downhill trend to the formation final product CH_3_OH and CH_3_OOH, representing a high electronic activity. The spontaneous adsorption of CH_4_ and facile C−H bond cleavage indicates the fast activation of C−H bond, which leads to the efficient DMC process. Particularly, the further reaction of CH_3_OOH toward HCOOH has been suppressed by the high energetic barrier of 1 eV, guaranteeing the reaction locking for desired products. The formation of CH_3_OH releases 2.87 eV, which is slightly larger energy than that of CH_3_OOH (2.53 eV), explaining the higher yield of oxygenates in our experiments. Meanwhile, the high energy cost (2.01 eV) to achieve further C−H bond cleavage of CH_3_ facilitates the high selectivity of DMC on the SAs Rh-CeO_2_ NWs (Fig. 4h). In contrast, the activation of C−H bond in CH_4_ faces stepped energy barriers toward ^*^CH_2_ on Rh/CeO_2_ NWs, indicating a much lower selectivity of oxygenates. The formation of CH_3_OOH induced by the ∙OOH radical requires an energy cost of 0.47 eV, while the generation of CH_3_OH is energetically favorable. Notably, both ^*^CH_2_ and CH_3_OOH exhibit an evident energy drop toward the formation of HCOOH, indicating that the overoxidation of CH_4_ is preferred. However, the further oxidation of HCOOH to CO shows an energy barrier of 2.08 eV, which further lowers the DMC efficiency. The overall energy release is 1.92 eV, which is much smaller than that of SAs Rh-CeO_2_ NWs, supporting the stronger reaction activity of SAs Rh-CeO_2_ NWs toward the DMC conversion (Fig. 4i).

### Reaction pathways

Combining the results from EPR, in situ DRIFTS and DFT calculations, we thus summarized the reaction paths of DMC on the SAs Rh-CeO_2_ NWs and Rh/CeO_2_ NWs (Supplementary Figs. 13 and 14). As displayed in Supplementary Fig. 13, for the SAs Rh-CeO_2_ NWs, H_2_O_2_ can decompose into ∙OH on two neighboring Ce (III) atoms (step 1). On the other hand, H_2_O_2_ can decompose into ∙OOH on Ce (IV), and the remaining ∙H will combine with [O] (lattice O of CeO_2_) connected with the Ce (IV) in CeO_2_ NWs to form ∙[O]H (step 2). The adsorbed CH_4_ on Rh SA can be oxidized into ∙CH_3_ and H_2_O with the assistance of ∙OH (step 3). As a result, the formed ∙CH_3_ will react with the ∙OOH and ∙[O]H radicals to produce CH_3_OOH and CH_3_[O]H via steps 4 and 5. It should be noted that the lattice [O] in CeO_2_ NWs will be compensated by H_2_O_2_ (Ce (III) → Ce (IV)). By contrast, the reaction paths are different on the Rh/CeO_2_ NWs, despite the paths of H_2_O_2_ decomposition (steps 1−2) are the same (Supplementary Fig. 14). On Ru clusters, CH_4_ can be activated into ∙CH_3_ by ∙OH, which may (i) react with ∙[O]H to CH_3_[O]H (step 4), or (ii) combine with ∙OOH to produce CH_3_OOH (step 5), or (iii) be further oxidized into ^*^CH_2_ by ∙OH (step 6). Both the CH_3_OOH and ^*^CH_2_ will be converted into ^*^CH_2_O (steps 7 and 8) and then to ^*^HCOOH on the Rh/CeO_2_ NWs (step 9). Finally, HCOOH will be further oxidized into CO_x_ in the presence of ∙OH (steps 10−11).

## Discussion

In summary, we demonstrated that SAs Rh-CeO_2_ NWs can be used for DMC to oxygenates. Compared with the low selectivity (56.4%) and yield (189.4 mmol g_Rh_^−1^ h^−1^) of oxygenates from Rh/CeO_2_ NWs, the selectivity and yield of CH_3_OH and CH_3_OOH are 93.9% and 1231.7 mmol g_Rh_^−1^ h^−1^ on SAs Rh-CeO_2_ NWs at 50 °C, which to the best of our knowledge outperforms the reported values in the literatures. In situ characterizations and experiments were performed to study the mechanism. It is shown that CeO_2_ NWs play a vital role in the generation of ∙OH and ∙OOH radicals, which can significantly promote the oxidation of CH_4_ via different reaction paths. To be more specific, SAs Rh-CeO_2_ NWs can selectively activate CH_4_ to ^*^CH_3_, leading to the formation of oxygenate in the presence of H_2_O_2_, while Rh/CeO_2_ NWs favor the overoxidation of CH_4_ to form CO_x_, leading to a low selectivity and yield of oxygenates. DFT calculations reveal the facile C−H bond activation and reaction locking on SAs Rh-CeO_2_ NWs guarantees the high selectivity and yield of DMC, supplying solid evidence for experimental results. This work may not only provide a highly active and selective catalyst for DMC to oxygenate but also promote the researches of SACs in heterogeneous catalysis.

## Methods

### Preparation of CeO_2_ nanowires (NWs) and SAs Rh-CeO_2_ NWs

In a typical preparation of CeO_2_ NWs, 12 mL CeCl_3_ (0.5 mmol, Alfa Aesar) and sodium oleate (0.75 mmol, Tokyo Chemical Industry) aqueous solution were added into a 20-mL Teflon-lined stainless-steel autoclave. The mixture was magnetically stirred at 800 rpm (round per minute) for 0.5 h. To this solution, *n*-butylamine (1 mL, Sinopharm) was slowly added into the solution under stirring for another 0.5 h. Afterward, the autoclave was heated at 160 °C for 10 h before it was cooled to room temperature. The resulting products were collected by centrifugation and washed three times with cyclohexane/acetone mixture, and then dried at room temperature overnight. The powder products were subjected to thermal annealing in an air atmosphere at 400 °C for 10 min at a heating rate of 10 °C min^−1^. For SAs Rh-CeO_2_ NWs, all the parameters were the same with CeO_2_ NWs, except for adding extra Na_3_RhCl_6_ (5 μmol, Aldrich).

### Preparation of Rh/CeO_2_ NWs and Rh/CeO_2_-com

The Rh/CeO_2_ NWs and Rh/CeO_2_-com were prepared via a conventional wet-impregnation method. Typically, a certain amount of Na_3_RhCl_6_ solution was dropwise added into the as-prepared CeO_2_ NWs and commercial CeO_2_ (99.9%, Inoke) under moderate stirring. The resultant slurry was mixed evenly and then dried in an oven at 80 °C overnight, and then subjected to thermal annealing in air at 400 °C for 10 min at a heating rate of 10 °C min^−1^. Finally, the calcinated samples were re-washed water for three times via centrifugation, and then dried at 100 °C overnight.

### Characterization

The morphologies and sizes of the NWs were determined by TEM (Hitachi, HT7700) at 120 kV. AC-HAADF-STEM images were taken on JEM-ARM200F with a cold-field emission gun and a spherical aberration corrector. The Rh loading amounts were determined by the inductively coupled plasma atomic emission spectroscopy (ICP-AES) (710-ES, Varian). XRD patterns were collected on X’Pert-Pro MPD diffractometer (Netherlands PANalytical) with a Cu Kα X-ray source (*λ* = 1.540598 Å). XPS was done with an SSI S-Probe XPS Spectrometer. The carbon peak at 284.6 eV was used as a reference to correct for charging effects. The X-ray absorption data at the Rh K-edge of the samples were recorded at room temperature in transmission mode using ion chambers at beamline BL14W1 of the Shanghai Synchrotron Radiation Facility (SSRF), China. The station was operated with a Si (311) double-crystal monochromator. During the measurement, the synchrotron was operated at energy of 3.5 GeV and a current between 150 and 210 mA. The photon energy was calibrated with the first inflection point of Rh K-edge in Rh metal foil.

### Typical process for selective oxidation of CH_4_

The selective oxidation of CH_4_ was performed in a 60-mL stainless-steel autoclave. Typically, 20 mL H_2_O_2_ solution (1 M) and 10 mg of catalyst were added into a Teflon inlet. Afterward, the autoclave was pressurized with CH_4_ (0.5 MPa). The reaction was performed at 50 °C with stirring at 800 rpm for 1 h. In our work, we used a commercial Agilent GC integrated system with a flame ionization detector (FID) for the detection of gaseous products. This system contains three columns (two hayesep Q columns and one 5 A mol sieve column) for the separation of gaseous products. Both CO and CO_2_ were converted into CH_4_ via a methanator to analyze by FID. All the gaseous products were introduced into two tandem hayesep Q columns for pre-separation. CO_2_ was completely separated in this process and introduced into the FID via a switch valve. The remaining gases were further introduced into a 5 A mol sieve column for further separation. ^1^H nuclear magnetic resonance spectroscopy (^1^H-NMR, Bruker 600 MHz) was employed for structural analysis of liquid products, in which dimethylsulfoxide (DMSO, 1‰) and deuteroxide were used as the internal standard and solvent, respectively. The liquid products were analyzed by a gas chromatograph (Persee G5) with a FID using a KB-5 column. In total, 10 μL of isopropanol/H_2_O (1%) was used as the internal standard adding into 1 mL reaction mixture for analyzing CH_3_OH and CH_3_OOH. The amount of CH_3_OH was calculated using the standard curve method. Afterward, CH_3_OOH was converted to CH_3_OH using hydrazine hydrate (0.1 mL). The total amount of CH_3_OH and CH_3_OOH were analyzed by gas chromatograph, and the amount of CH_3_OOH was obtained by minusing. For each catalytic test, error bar was obtained by repeating three times. The yields of products and the selectivity were calculated using Eqs. (1) and (2).

1 $${\mathrm{Yields}}\,{\mathrm{of}}\,{\mathrm{products}}\,\left( {{\mathrm{mmol}} \; {\mathrm{g}}_{{\mathrm{Rh}}}^{{\mathrm{ - 1}}} \; {\mathrm{h}}^{{\mathrm{ - 1}}}} \right) \!= {\mathrm{mmol}}\,{\mathrm{of}}\,{\mathrm{products/g}}\,{\mathrm{of}}\;{\mathrm{Rh/h}}\,{\mathrm{of}}\,{\mathrm{reaction}}\,{\mathrm{time}}$$

2 $${\mathrm{Oxygenates}}\,{\mathrm{selectivity}}\,\left( \% \right) = 	\,\,{\mathrm{mmol}}\,{\mathrm{of}}\,\left( {{\mathrm{CH}}_{\mathrm{3}}{\mathrm{OOH}}\; +\; {\mathrm{CH}}_{\mathrm{3}}{\mathrm{OH}}} \right) \\ 	\times 100/{\mathrm{mmol}}\,{\mathrm{of}}\,{\mathrm{all}}\,{\mathrm{products}}$$

### EPR measurement

The detection of free radicals in the reaction process of DMC was performed at a JEOL JES-FA200 electron paramagnetic resonance spectroscopy (EPR, 9.062 GHz), using DMPO as the scavenger. Typically, 1 mL DMPO-H_2_O (100 mmol L^−1^) was added into 1 mL of reaction mixture. The mixed solution was immediately transferred to a capillary tube (diameter: 0.1 mm; filling liquid height: ~5 cm), which was then fixed in the resonant cavity of the spectrometer. EPR measurements were detected at room temperature and recorded by three scans. The contrast experiments were performed to determine the type of radicals by characteristic peaks. Contrast experiment 1 (labeled as DMPO + H_2_O_2_ + Fe^2+^): 1 mL ferrous (II) sulfate (FeSO_4_, 50 mmol L^−1^) and nitric acid solution (pH = 4) were mixed with 1 mL DMPO-H_2_O (100 mmol L^−1^) under moderate stirring for 2 min, followed by adding 50 μL of H_2_O_2_ (30 wt.%). Contrast experiment 2 (labeled as DMPO + H_2_O_2_ + Fe^2+^ + CH_3_OH): all the processes were the same with contrast experiment 1, except for the addition of 1 mL of CH_3_OH (100 mmol L^−1^) into the solution.

### CO-DRIFTS measurement

In all, 20 mg sample was packed into a Harrick Praying Mantis high-temperature reaction chamber (CaF_2_ windows) mounted inside of a Thermo Scientific Praying Mantis diffuse reflectance adapter, set inside of a Thermo Scientific Nicolet 6700 Fourier transform infrared (FT-IR) spectrometer using liquid nitrogen cooled mercury–cadmium–telluride detector (MCT). Gases were flowed to the reaction chamber using Alicat mass flow controllers. Prior to CO-DRIFTS measurement, the sample was pretreated by heating in CO/Ar (10 vol.%) flow (50 mL min^−1^) at different temperatures (e.g., −30 °C, 50 °C, and 150 °C), followed by flushing with Ar flow (50 mL min^−1^) for 1 h. After cooling to −30 °C in the same Ar flow, CO/Ar (10 vol.%, 50 mL min^−1^) was flowed through the sample at −30 °C for 0.5 h. Finally, the sample was flushed with Ar flow (50 mL min^−1^) at −30 °C for 0.5 h to remove the physically adsorbed CO on the surface sample. The CO-DRIFTS spectra were obtained by averaging 16 sequentially collected scans at a resolution of 4 cm^−1^.

### In situ CH_4_-DRIFTS measurement

For in situ CH_4_-DRIFT, 20 mg sample was packed into a Harrick Praying Mantis high-temperature reaction chamber (CaF_2_ windows) mounted inside of a Thermo Scientific Praying Mantis diffuse reflectance adapter, set inside of a Thermo Scientific Nicolet 6700 FT-IR spectrometer using liquid nitrogen cooled MCT detector. The sample was pretreated by heating in Ar flow (50 mL min^−1^) at 400 °C for 1 h and cooled down to 50 °C in the same Ar flow. Gases (5 vol.% CH_4_/Ar or Ar) were flowed using Alicat mass flow controllers. The hydrogen peroxide is brought into the chamber by a carrier gas (5 vol.% CH_4_/Ar) with a positive pressure (0.15 MPa). Afterward, 5 vol.% CH_4_/Ar with the hydrogen peroxide was flowed through the catalyst bed at 50 °C for 0.5 h, followed by flushing with Ar flow (50 mL min^−1^) for 0.5 h at the same temperature. The in situ CH_4_-DRIFTS spectra were obtained by averaging 16 sequentially collected scans at a resolution of 4 cm^−1^. For CH_4_-DRIFTS measurement, all the parameters are the same with in situ CH_4_-DRIFTS measurement, except for the absence of H_2_O_2_.

### DFT calculations

All calculations were carried out using DFT implemented in the CASTEP^36^. The electron exchange and correlation interaction were calculated with the generalized gradient approximation (GGA) in the parametrization of Perdew–Burke–Ernzerhof (PBE) pseudopotentials^37,38^. To minimize the computational cost for the surface modeling, ultrasoft pseudopotential scheme has been chosen with the cutoff energy of 410 eV^39,40^. For both SAs Rh-CeO_2_ and Rh/CeO_2_, the CeO_2_ crystal has been cleaved along the (111) plane with three-layer thickness. For SAs Rh-CeO_2_ model, only one Rh atom is deposited onto the CeO_2_ surface within the unit cell to demonstrate the single-atom catalyst. For Rh/CeO_2_ NWs model, the Rh cluster consists of eight atoms, which is cleaved from the bulk Rh crystal along the (111) plane. We imposed a 15 Å vacuum space along *z*-direction to supply sufficient space for the adsorption behaviors without interactions between lattice. For all the geometry optimizations, the Hellmann–Feynman forces will be converged to <0.001 eV/A, while the total energy has been converged to 5 × 10^−5^ eV per atom. The coarse k-point has been applied for the energy minimization based on the Broyden–Fletcher–Goldfarb–Shannon (BFGS) algorithm^41,42^.

## Supplementary information

Supplementary Information

## Data Availability

The data supporting this study are available in the paper and  Supplementary Information. All other relevant source data are available from the corresponding authors upon reasonable request.
