# Supplementary material for: High-efficiency direct methane conversion to oxygenates on a cerium dioxide nanowires supported rhodium single-atom catalyst
Source: Nat Commun. 2020 Feb 19;11:954. doi: 10.1038/s41467-020-14742-x (PMC7031227; doi:10.1038/s41467-020-14742-x)
Supplement: Supplementary file 1 — Supplementary Information [file 41467_2020_14742_MOESM1_ESM.pdf]

## Supplementary Information

# High-efficiency direct methane conversion to oxygenates on a cerium dioxide nanowires supported rhodium single-atom catalyst

Bai *et al.*

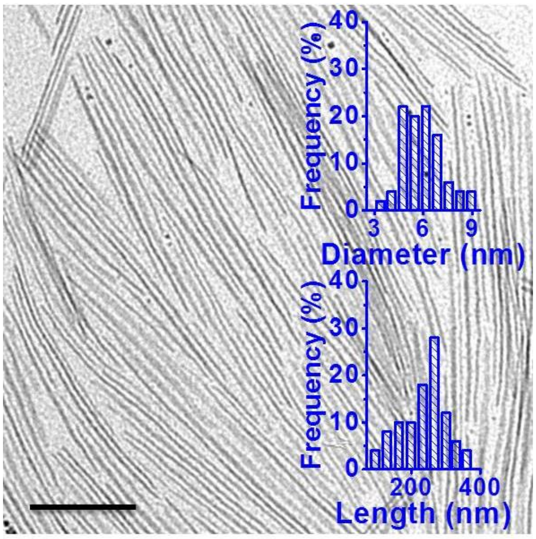


**Supplementary Fig. 1. TEM image of the $CeO_2$ NWs.** Inset shows the histograms on the diameter and length of $CeO_2$ NWs. The scale bar is 100 nm.

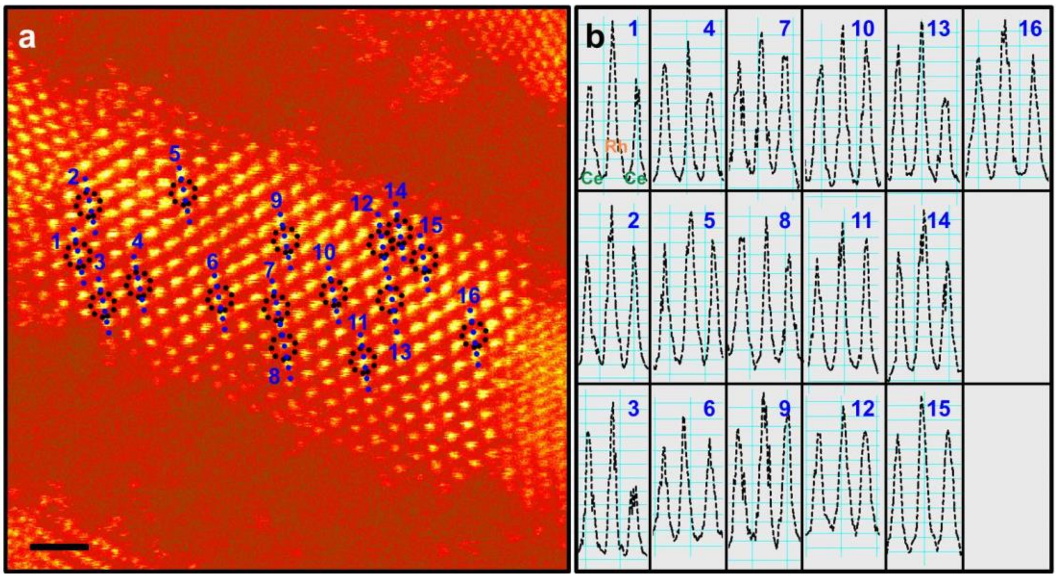


**Supplementary Fig. 2. Structural analyses of SAs Rh-$CeO_2$ NWs.** (**a**) AC-HAADF/STEM image in temperature color of SAs Rh-$CeO_2$ NWs. The isolated Rh atoms are marked with black circles. (**b**) The corresponding intensity profiles marked by blue dash line from the HADDF-STEM in temperature-color image in (**a**). The scale bar in (**a**) is 1 nm.

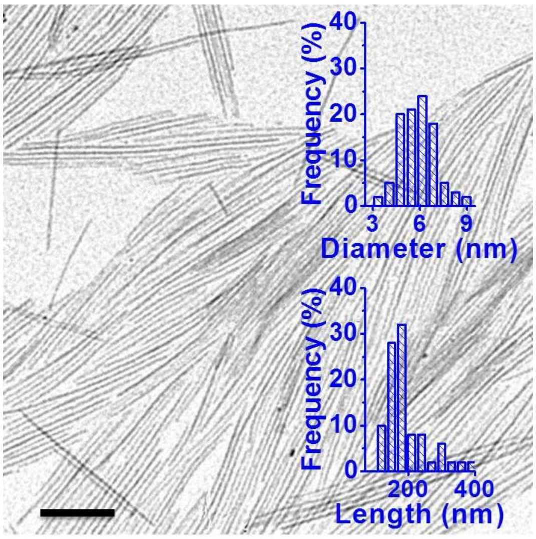


**Supplementary Fig. 3. TEM image of the Rh/$CeO_2$ NWs.** Inset shows the histograms on the diameter and length of Rh/$CeO_2$ NWs. The scale bar is 100 nm.

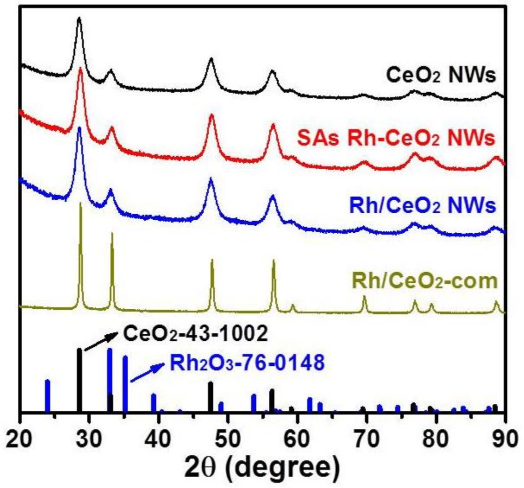


**Supplementary Fig. 4. XRD patterns of $CeO_2$ NWs, SAs Rh-$CeO_2$ NWs, Rh/$CeO_2$ NWs and Rh/$CeO_2$-com.** The feature peaks of $CeO_2$ and $Rh_2O_3$ were given in the XRD pattern.

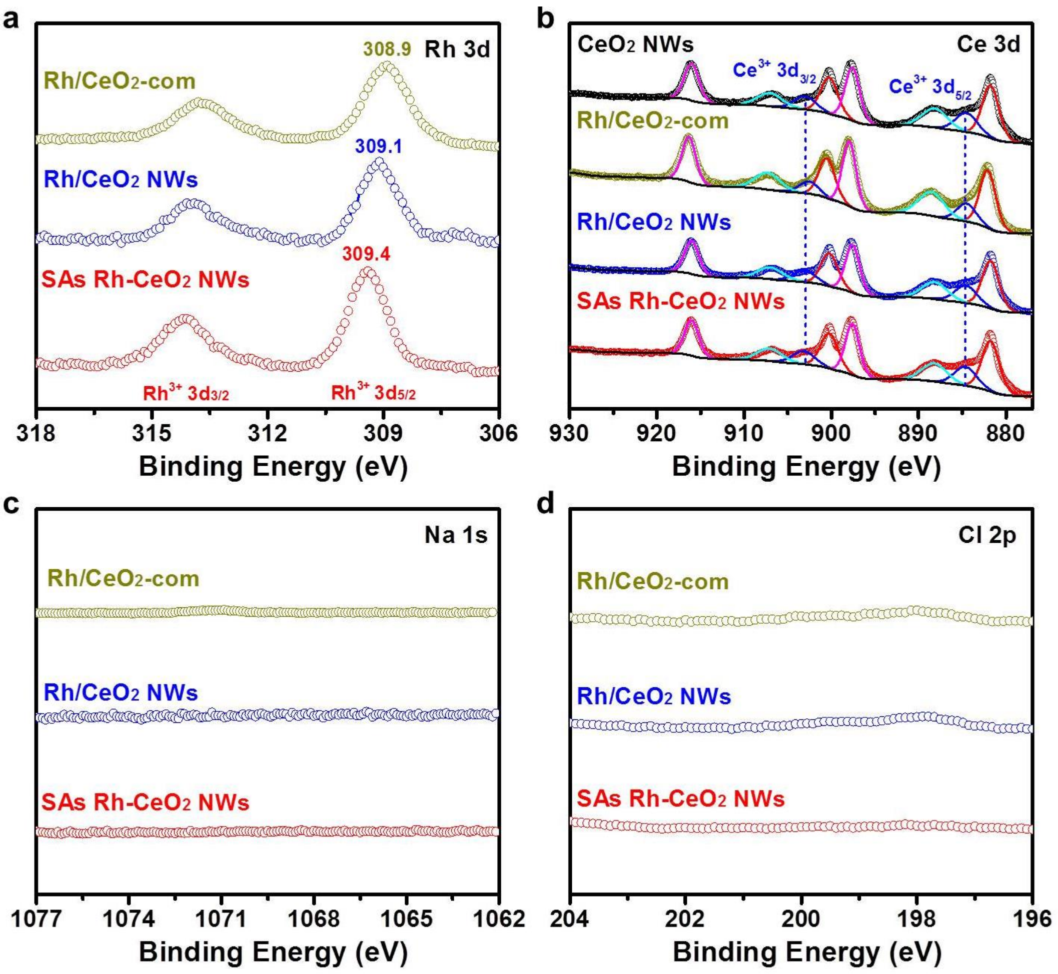


**Supplementary Fig. 5. XPS spectra of Rh/$CeO_2$-com, $CeO_2$ NWs, Rh/$CeO_2$ NWs, and SAs Rh-$CeO_2$ NWs.** (**a**) Rh 3d, (**b**) Ce 3d, (**c**) Na 1s, and (**d**) Cl 2p XPS spectra.

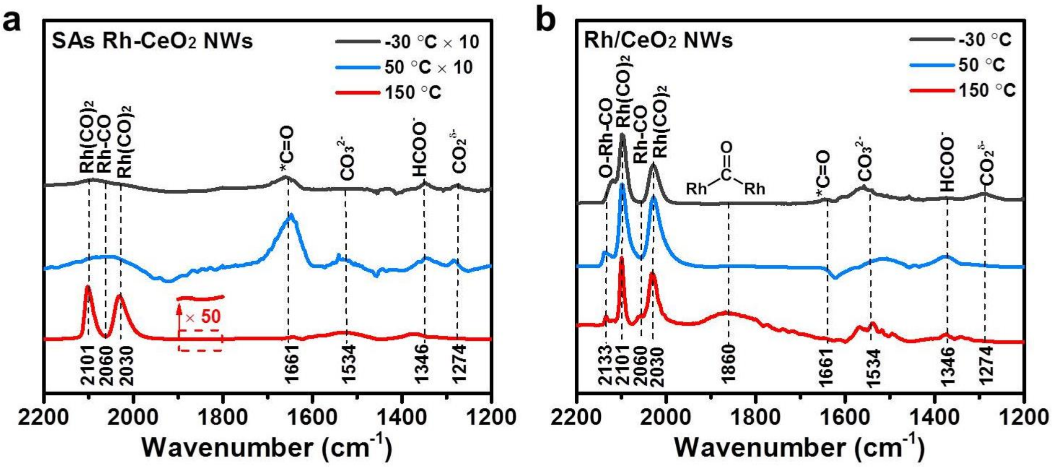


**Supplementary Fig. 6. CO-DRIFTS spectra of SAs Rh-$CeO_2$ NWs and Rh/$CeO_2$ NWs.** (**a**) SAs Rh-$CeO_2$ NWs and (**b**) Rh/$CeO_2$ NWs. The CO-DRIFTS spectra were collected at -30 ℃, 50 ℃, and 150 ℃, respectively.

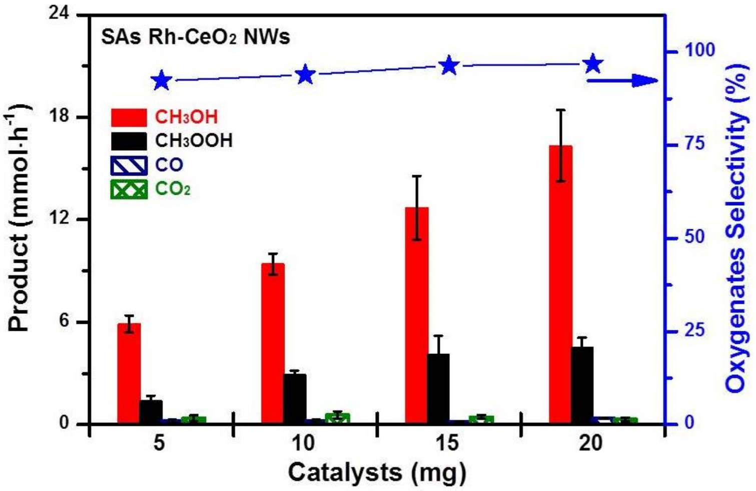


**Supplementary Fig. 7. DMC performance on different amount of SAs Rh-CeO$_2$ NWs.** Reaction conditions: $P_{CH4}$:0.5 MPa, $H_2O_2$: 20 mL (1 M), $T$: 50 ℃, reaction time: 1 h.

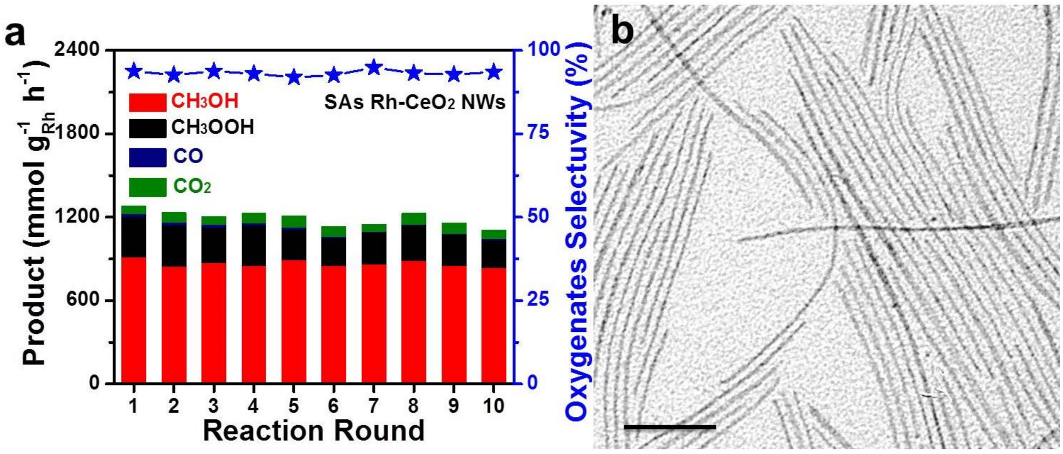


**Supplementary Fig. 8. Stability test of SAs Rh-CeO$_2$ NWs for DMC in ten cycles**. (**a**) The yield of products and selectivity of oxygenates. (**b**) TEM image of SAs Rh-CeO$_2$ NWs after ten DMC cycles. Reaction conditions: $P_{CH4}$: 0.5 MPa, $H_2O_2$: 20 mL (1 M), $T$: 50 ℃, reaction time: 1 h, and catalyst weight: 10 mg. The scale bar in (**b**) is 50 nm.

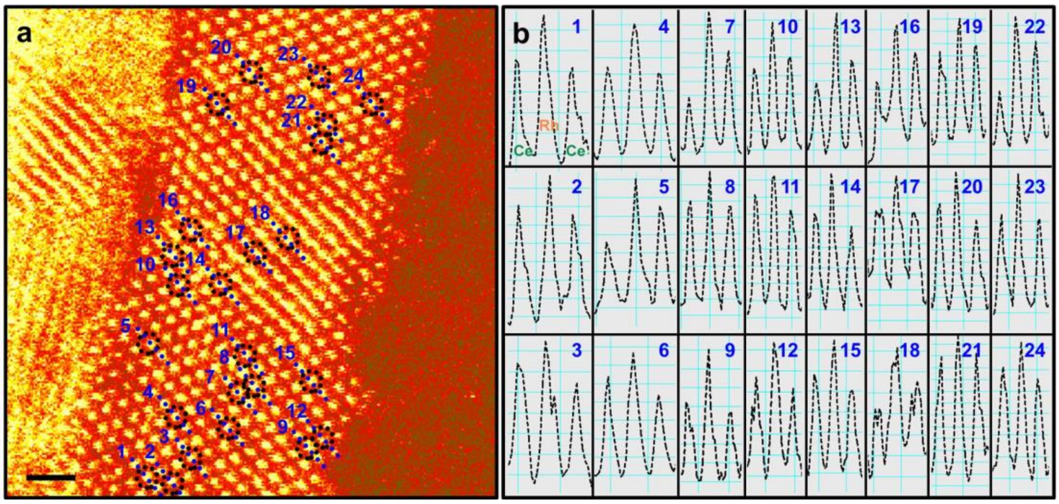


**Supplementary Fig. 9. Structural analyses of used SAs Rh-$CeO_2$ NWs.** (**a**) AC-HAADF/STEM image in temperature color of SAs Rh-$CeO_2$ NWs after 10 DMC cycles, (**b**) The corresponding intensity profiles marked by blue dash line from the HADDF-STEM in temperature-color image in (**a**). The scale bar in (**a**) is 1 nm.

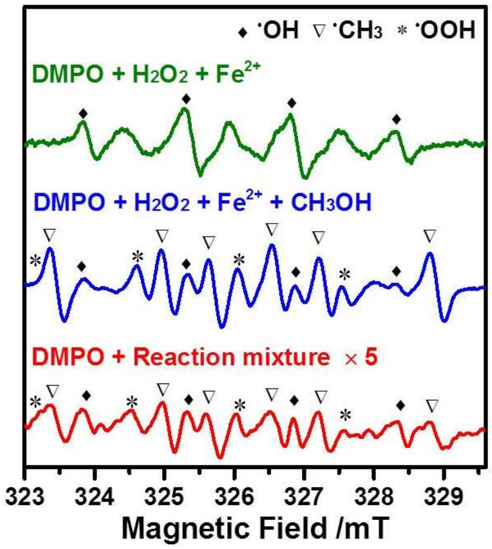


**Supplementary Fig. 10. EPR spectra of SAs Rh-$CeO_2$ NWs in different systems.** DMPO was used as radical scavenger, which was added into the reaction systems of $H_2O_2$ + $Fe^{2+}$ (green), $H_2O_2$ + $Fe^{2+}$ + $CH_3OH$ (blue) and reaction system (red).

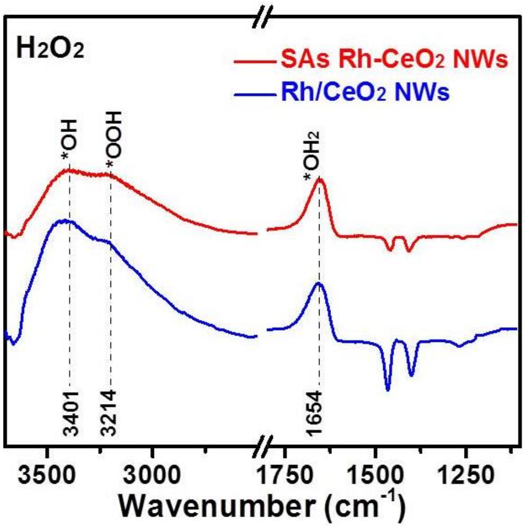


**Supplementary Fig. 11. DRIFTS spectra of SAs Rh-CeO$_2$ NWs and Rh/CeO$_2$ NWs after exposing to H$_2$O$_2$.** The DRIFTS spectra were collected at 50 ℃.

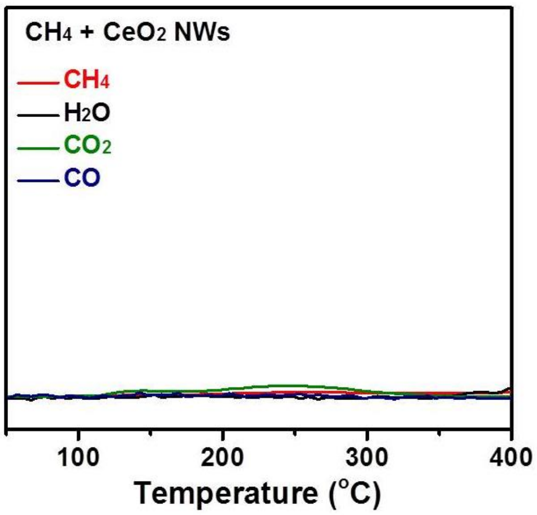


**Supplementary Fig. 12. $CH_4$-TPSR measurement on the $CeO_2$ NWs.** The weak peaks observed in the $CH_4$-TPSR curves indicate that $CeO_2$ NWs are inactive for DMC under the indicated conditions.

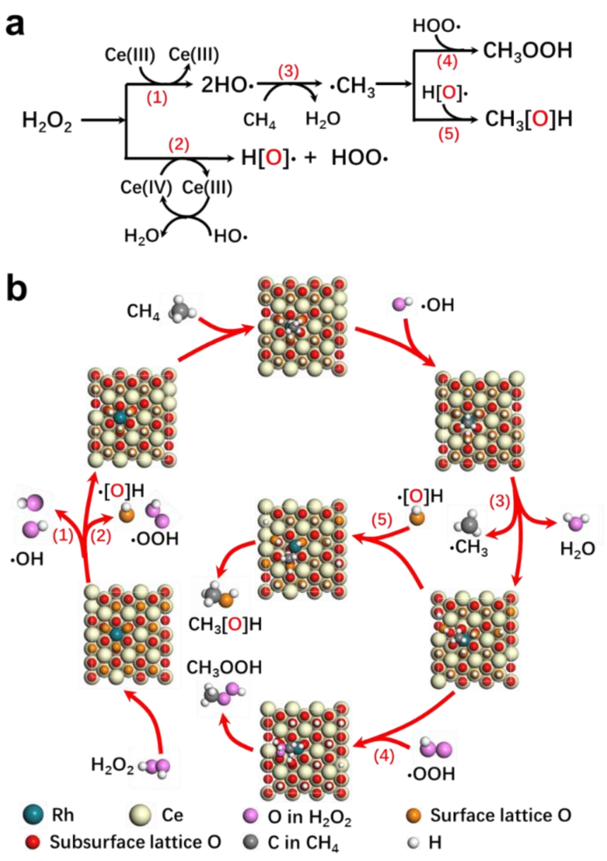


**Supplementary Fig. 13. Schematic illustration of DMC on SAs Rh-CeO$_2$ NWs.** (**a**) Scheme of DMC on SAs Rh-CeO$_2$ NWs in the presence of $H_2O_2$, (**b**) reaction paths of DMC on SAs Rh-CeO$_2$ NWs.

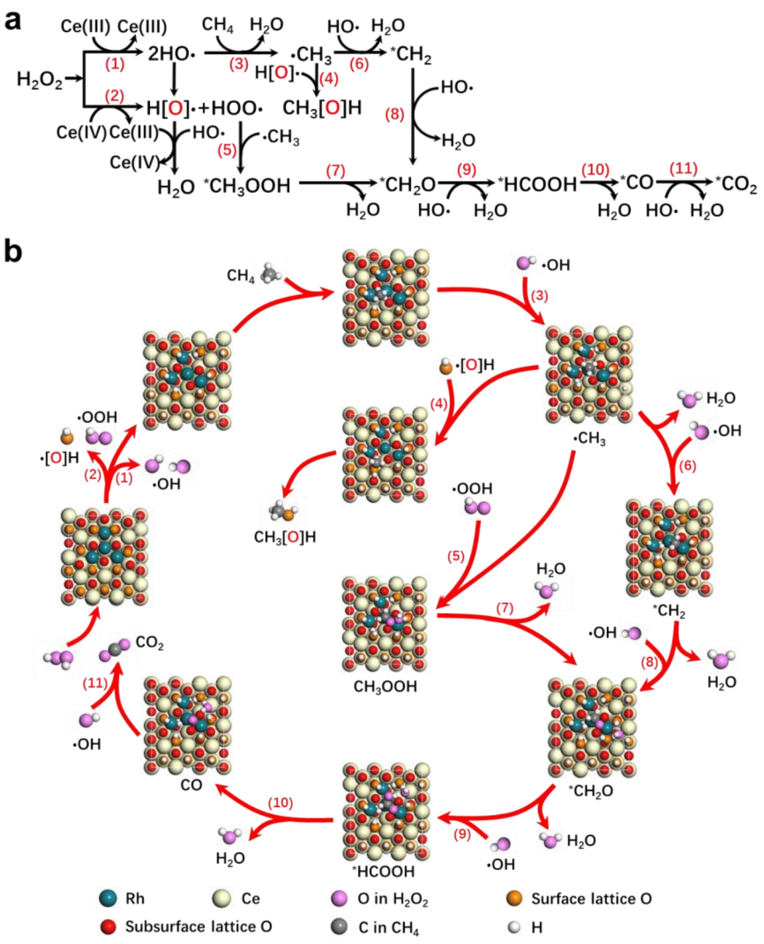


**Supplementary Fig. 14. Schematic illustration of DMC on Rh/CeO$_2$ NWs.** (**a**) Scheme of DMC on Rh/CeO$_2$ NWs in the presence of $H_2O_2$, (**b**) reaction paths of DMC on Rh/CeO$_2$ NWs.

**Supplementary Table 1.** Physiochemical properties of different samples.

| Sample | Length (nm) | Diameter (nm) | Rh content (wt%)[a] |
|---|---|---|---|
| $CeO_2$ NWs | 260 ± 100 | 6.2 ± 2.7 | - |
| SAs Rh-$CeO_2$ NWs | 240 ± 120 | 5.7 ± 2.7 | 0.29 |
| Rh/$CeO_2$ NWs | 180 ± 110 | 6.2 ± 2.7 | 0.30 |
| Rh/$CeO_2$-com | – | – | 0.31 |

[a] The contents of Rh were measured by ICP-AES.

**Supplementary Table 2.** The comparison of DMC performance over SAs Rh-$CeO_2$ NWs and other reported catalysts.

| Catalyst | Reaction condition | | | Products ($mmol \cdot g_{metal}^{-1} \cdot h^{-1}$) | | | Oxygenates selectivity (%) | Ref. |
|---|---|---|---|---|---|---|---|---|
| | Reactants | $T$ (°C) | $t$ (h) | $CH_3OH$ | $CH_3OOH$ | $CH_3COOH$ | | |
| 0.29 wt. % Rh-$CeO_2$ SAs | $P_{CH4}$=0.5 MPa; $C_{H2O2}$=1 mol $L^{-1}$; Cat.: 10 mg | 50 | 1 | 940.3 | 291.4 | - | 93.9 | This work |
| 1 wt. % AuPd/$TiO_2$ | $P_{CH4}$=3.05 MPa; $C_{H2O2}$=0.5 mol $L^{-1}$; Cat.: 10 mg | 70 | 0.5 | 2.9 | 11.2 | - | 90.0 | 1 |
| 0.3 wt. % Rh/$ZrO_2$ SAC | $P_{CH4}$=3.0 MPa; $C_{H2O2}$=0.5 mol $L^{-1}$; Cat.: 150 mg | 70 | 1 | 10.3 | 2.3 | - | 78.4 | 2 |
| 2 wt. % CuO + 0.01 wt.% Pd/ZSM-5 | $P_{CH4}$=3.0 MPa; $C_{H2O2}$=0.5 mol $L^{-1}$; Cat.: 28 mg | 95 | 0.5 | 195.7 | 0.4 | - | 86.4 | 3 |
| 5 wt.% AuPd/$TiO_2$ | $P_{CH4}$=2.3 MPa; $P_{O2}$=0.05 MPa; $P_{H2}$=0.026 MPa; Cat.: 27.6 mg | 50 | 0.5 | 1.9 | 0.4 | - | 83.3 | 4 |
| AuPd colloid | $P_{CH4}$=3.0 MPa; $P_{O2}$=0.5 MPa; $C_{H2O2}$=0.1 mol $L^{-1}$; Cat.: 6.6 μmol | 50 | 0.5 | 15.2 | 34.8 | - | 88.0 | 5 |
| 0.5 wt. % Rh-ZSM-5 | $P_{CH4}$=2.0 MPa; $P_{O2}$=0.2 MPa; $P_{CO}$=0.5 MPa; Cat.: 20 mg | 150 | 3 | 137.9 | - | 1419.7 | 85.8 | 6 |
| 0.1 wt. % Rh/ZSM-5 | $P_{CH4}$=5.0 MPa; $P_{O2}$=0.8 MPa; $P_{CO}$=1.0 MPa; Cat.: 28 mg | 150 | 12 | 244.1 | - | 2500.0 | - | 7 |

**Supplementary Table 3.** DMC performance of SAs Rh-$CeO_2$ NWs using different oxidants.

| **Catalyst** | **$H_2O_2$ (μmol)** | **$O_2$ (MPa)** | **Products ($mmol\cdot g_{Rh}^{-1}\cdot h^{-1}$)** | | | **Oxygenates selectivity (%)** |
|---|---|---|---|---|---|---|
| | | | $CH_3OH$ | $CH_3OOH$ | $CO_x$ | |
| SAs Rh-$CeO_2$ NWs | 0 | 0.5 | 14.5 | 3.1 | 281.7 | 5.9 |
| SAs Rh-$CeO_2$ NWs | 10 | 0.5 | 929.7 | 229.7 | 89.7 | 92.8 |

## Supplementary References

1. Williams, C. et al. Selective oxidation of methane to methanol using supported AuPd catalysts prepared by stabilizer-free sol-immobilization. *ACS Catal.* **8**, 2567−2576 (2018).

2. Kwon, Y. et al. Selective activation of methane on single-atom catalyst of rhodium dispersed on zirconia for direct conversion. *J. Am. Chem. Soc.* **139**, 17694−17699 (2017).

3. Huang, W. X. et al. Low-temperature transformation of methane to methanol on $Pd_1O_4$ single sites anchored on the internal surface of microporous silicate. *Angew. Chem. Int. Ed.* **55**, 13441−13445 (2016).

4. Ab Rahim, M. H. et al. Oxidation of methane to methanol with hydrogen peroxide using supported gold-palladium alloy nanoparticles. *Angew. Chem. Int. Ed.* **52**, 1280−1284 (2013).

5. Agarwal, N. et al. Aqueous Au-Pd colloids catalyze selective $CH_4$ oxidation to $CH_3OH$ with $O_2$ under mild conditions. *Science* **358**, 223−227 (2017).

6. Shan, J. et al. Mild oxidation of methane to methanol or acetic acid on supported isolated rhodium catalysts. *Nature* **551**, 605–608 (2017).

7. Tang, Y. Single rhodium atoms anchored in micropores for efficient transformation of methane under mild conditions. *Nat. Commun.* **9**, 1231 (2018)
